# *In Silico* Discovery of Plant-Origin Natural Product Inhibitors of Tumor Necrosis Factor (TNF) and Receptor Activator of NF-κB Ligand (RANKL)

**DOI:** 10.3389/fphar.2018.00800

**Published:** 2018-07-25

**Authors:** Georgia Melagraki, Evangelos Ntougkos, Dimitra Papadopoulou, Vagelis Rinotas, Georgios Leonis, Eleni Douni, Antreas Afantitis, George Kollias

**Affiliations:** ^1^Hellenic Military Academy, Vari, Greece; ^2^Division of Immunology Biomedical Sciences Research Center “Alexander Fleming,”, Vari, Greece; ^3^Department of Experimental Physiology, Medical School, National and Kapodistrian University of Athens, Athens, Greece; ^4^Department of Biotechnology, Agricultural University of Athens, Athens, Greece; ^5^NovaMechanics Ltd., Nicosia, Cyprus

**Keywords:** direct TNF inhibitors, RANKL inhibitors, natural products, autoimmune diseases, virtual screening, molecular dynamics

## Abstract

An *in silico* drug discovery pipeline for the virtual screening of plant-origin natural products (NPs) was developed to explore new direct inhibitors of TNF and its close relative receptor activator of nuclear factor kappa-B ligand (RANKL), both representing attractive therapeutic targets for many chronic inflammatory conditions. Direct TNF inhibition through identification of potent small molecules is a highly desired goal; however, it is often hampered by severe limitations. Our approach yielded a priority list of 15 NPs as potential direct TNF inhibitors that were subsequently tested *in vitro* against TNF and RANKL. We thus identified two potent direct inhibitors of TNF function with low micromolar IC_50_ values and minimal toxicity even at high concentrations. Most importantly, one of them (A11) was proved to be a dual inhibitor of both TNF and RANKL. Extended molecular dynamics simulations with the fully automated EnalosMD suite rationalized the mode of action of the compounds at the molecular level. To our knowledge, these compounds constitute the first NP TNF inhibitors, one of which being the first NP small-molecule dual inhibitor of TNF and RANKL, and could serve as lead compounds for the development of novel treatments for inflammatory and autoimmune diseases.

## Introduction

Tumor necrosis factor (TNF) is an important human cytokine (Beutler et al., 1985) that is involved in a number of critical biological processes and diseases, including rheumatoid arthritis, Crohn’s disease, multiple sclerosis, inflammatory bowel disease, psoriatic arthritis, AIDS, and cancer (Kollias et al., 1999; Apostolaki et al., 2010). Disruption of TNF binding to its principal receptor, TNFR1, has been a long-desired goal in the development of novel autoimmune therapeutics (Douni and Kollias, 1998; Kollias and Kontoyiannis, 2002). Previous *in vivo* studies from our group demonstrated that deregulated TNF production induces chronic polyarthritis in a transgenic animal model and the disease could be treated by proper anti-TNF therapy (Keffer et al., 1991). These research efforts were vital in directing the attention of the pharmaceutical industry to initial anti-TNF approaches, which eventually resulted in clinical trials that were successfully performed for a variety of chronic inflammatory diseases, including rheumatoid arthritis (Elliott et al., 1993), psoriasis, psoriatic arthritis, Crohn’s disease, juvenile idiopathic arthritis, spondyloarthritis, and Behçet’s disease (Sfikakis, 2010).

To date, three synthetic antibodies that block the activity of TNF have been reported, namely infliximab, adalimumab, and etanercept (Olsen and Stein, 2004). However, these expensive agents are frequently used as secondary options for patients with a poor response to regular anti-rheumatic drugs (Chaudhari et al., 2016). Moreover, biologics are associated with several other drawbacks, including high cost, inadequate clinical response, need of intravenous administration, as well as increased risk of tuberculosis and hepatitis B due to the lowered immune response. Therefore, there is a clear need for orally available, well-tolerated, inexpensive drugs that block the production of TNF associated with pathological inflammation in rheumatoid arthritis and related conditions. It has been shown that the use of small molecules in direct TNF inhibition represents an attractive alternative that offers significant benefits, such as oral administration, shorter half-lives with reduced immunosuppression, and easier manufacturing at a lower cost (Sfikakis, 2010; Lo et al., 2017; Melagraki et al., 2018).

According to a recent report (Chaudhari et al., 2016), there are no late-stage rheumatoid arthritis products targeting TNF under development. Particularly, small molecule direct inhibition of protein–protein interactions (PPIs), such as the one between TNF and its receptor, is a nontrivial approach in drug development (Sackett and Sept, 2009; Wilson, 2009; David, 2012; Arkin et al., 2014). For this purpose, successful drug design requires the identification of compounds with low molecular weight, something extremely challenging, especially when attempting to block interactions between large molecules such as proteins (Lo et al., 2017). The successful recognition of small-molecule inhibitors is also hampered by the difficulty to identify potential “hot spots” as unique binding targets that are crucial for the disruption of biomolecular interactions.

Protein–protein interactions interfaces are mostly flat, extended (approximately 1,500–2,000 Å^2^), solvent-exposed, and are characterized by hydrophobic and electrostatic interactions (Jones and Thornton, 1996; Hwang et al., 2010; Sheng et al., 2015). The main difference between PPI interfaces and deep protein cavities, which usually bind small molecules, is their size, with the latter occupying a relatively small area of less than 500 Å^2^ (Fuller et al., 2009). Studies on the binding energy distributions over protein interfaces by mutational analyses demonstrated that only specific residues (hot spots) at the PPI interface contribute most of the binding energy, while the majority of PPI-interface residues are not important (Arkin and Wells, 2004). It was shown that hot spots rather assemble at the middle of the interface, to form a hydrophobic region similar in size to a small molecule, and possess conformational flexibility. The location of hot spots usually coincides with the putative binding sites of the protein, and these sites consist of a number of surface residues, which favorably contribute to small-molecule binding and are also critical in stabilizing PPIs. It has been shown that among all protein residues, these hot-spot regions contribute the major part of the binding energy in a protein–inhibitor complex. Therefore, successful identification of hot spots may offer significant advancements in the rational design of inhibitors (Kozakov et al., 2015a,b).

However, little progress has been obtained regarding fast and reliable identification of hot spots despite recent advances in high-throughput methodologies (Kouadio et al., 2005; Bakail and Ochsenbein, 2016). Various computational approaches for the recognition of hot spot areas have been developed by several research groups and include methodologies that employ dedicated energy functions (e.g., Rosetta, FoldX, and PCRPi) (Guerois et al., 2002; Kortemme et al., 2004; Guharoy et al., 2011), molecular simulations (Rajamani et al., 2004), computational alanine scanning (Kollman et al., 2000), and machine learning approaches [for instance, HSpred (Lise et al., 2011) and HotPoint (Tuncbag et al., 2010)].

Despite that PPIs vary in size and shape, the majority of inhibitors usually bind to hot spot regions that are restricted to small binding sites (<1000 Å^2^) (Smith and Gestwicki, 2012; Basse et al., 2013) and partner proteins are defined by short residue sequences at the interface (Perkins et al., 2010; London et al., 2013). An effective PPI inhibitor must possess a large surface area and participate in many hydrophobic interactions with the receptor. However, such a ligand is usually accompanied by high molecular weight and low solubility; therefore, various pharmacokinetic problems may arise (Sheng et al., 2015). Moreover, identifying an adequate starting structure for successful design of small-molecule PPI inhibitors is often hampered by the lack of information about natural PPI inhibitors. To date, most of the published small molecules are indirectly targeting TNF by downregulating its expression and only a limited number of compounds is reported to directly disrupt this interaction. These include the polysulfonated naphthylurea suramin and its analogs (Alzani et al., 1993; Mancini et al., 1999) and the indole-linked chromone SPD304 (He et al., 2005), the use of which is hampered by low potency and poor selectivity with a concomitant tendency to cause adverse effects (suramin) (McGeary et al., 2008), and cell toxicity (SPD304) (Sun and Yost, 2008). Moreover, Chan et al. (2010) identified two natural product (NP)-like molecules, two FDA-approved drugs, namely darifenacin and ezetimibe (Leung et al., 2011), and a metal-based iridium(III) biquinoline complex (Leung et al., 2012), which act as direct inhibitors of TNF. Recently, our group with the aid of cheminformatics techniques identified two additional small molecules (T23 and T8) that were shown to directly inhibit TNF function (Melagraki et al., 2017). Importantly, the above compounds were also potent against receptor activator of nuclear factor kappa-B ligand (RANKL) and presented low toxicity. In 2017, another TNF small-molecule inhibitor, JNJ525, was discovered by Blevitt et al. (2017). The mechanism of PPI disruption was attributed to a change in the quaternary structure of the protein by an aggregate conglomerate of JNJ525 in a way that TNFR1 binding to TNF is blocked.

Drug discovery based on NP-like scaffolds has rapidly advanced through novel computational approaches (Baig et al., 2016; Rodrigues et al., 2016). Recent developments have demonstrated the power of computationally treating complex NP structures to recognize their protein targets and to find specific applications in rational drug design (Reutlinger et al., 2014; Rodrigues et al., 2016; Basith et al., 2018; Lima et al., 2018; Zheng et al., 2018). The abundance of NPs or compounds inspired by NPs as drugs and drug candidates (Lesney, 2004) motivated us to search for novel TNF inhibitors among them. Given the high priority of plant-origin NPs in previous and current drug development efforts (including the terpenoids, e.g., Taxol and steroids, the glycosides, e.g., digitalis and the various flavonoids, and the alkaloids, e.g., camptothecins and the opiates), we focused on identifying novel TNF small molecule inhibitors from plant sources.

## Materials and Methods

In search of plant-origin NPs as direct TNF inhibitors, we combined chemoinformatics techniques, high-throughput virtual screening, and molecular dynamics (MD) simulations with experimental evaluation, ultimately aiming at discovering potent TNF-functioning NP inhibitors. 3,573 pure NPs of plant origin were virtually screened from the MEGxp database, which is one of the largest chemical libraries of NPs available (AnalytiCon Discovery); the highest scoring compounds were then tested *in vitro* to assess their inhibitory activity against TNF.

**SCHEME 1:**
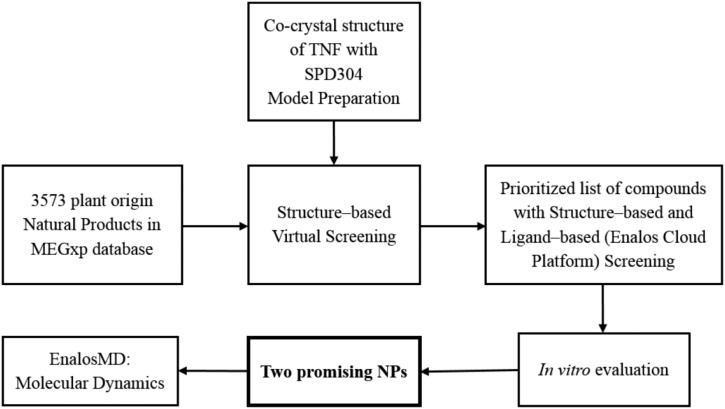
Strategy for the identification of NP TNF inhibitors.

Our strategy for identifying these novel plant-origin small molecule TNF inhibitors is presented in **Scheme 1**.

### Molecular Modeling

The initial model of TNF was built from the X-ray co-crystal structure of TNF dimer with SPD304 (PDB code: 2AZ5). All structures were prepared using Molegro’s Molecules and Protein Preparation Wizard (Thomsen and Christensen, 2006). Proper bond assignments, bond orders, hybridization, and charges were calculated by Molegro Virtual Docker (MVD) software (version-5.0) (Thomsen and Christensen, 2006). Explicit hydrogen atoms were added and their hydrogen bonding (HB) patterns were also determined by MVD. Since the 3D conformation of SPD304 is known from crystallographic data, a docking template was defined. SPD304 was replaced by each ligand in TNF, and template alignment considered ligands as fully flexible: the docking algorithm recognized the optimal conformation of the ligand when fitting to the template. The MolDock score (GRID) was used as a grid-based scoring function which pre-calculates potential energy values on an evenly spaced cubic grid in order to speed up calculations. A grid resolution of 0.30 Å was set to initiate the docking process and the binding site of the protein was defined to occupy the region surrounding SPD304 in the crystal structure (including residues Ser60, Gln61, Gly121, Tyr151, and Ala156). For the pose generation, the default setting was applied (MolDock SE), namely a maximum of 1500 iterations combined with a population size of 50. If the generated pose has an energy below the predefined energy threshold (100.0 in our study), it is included into the initial population for the “simplex evolution” algorithm (Thomsen and Christensen, 2006). This algorithm performs a combined local/global search on the poses generated by the pose generator. The number of the maximum iterations of the simplex evolution algorithm (Nelder–Mead simplex minimization) was set to 300 while the neighbor distance factor, the factor which determines how close the point of the initial simplex will be to the other randomly selected individuals in the population, was set to 1.0 (causes the initial simplex to span the neighbor points evenly).

### *In Vitro* Testing of TNF Inhibitors

Experiments included a TNF-induced death assay in L929 cells, a measurement of cytotoxicity in L929 cells, and a TNF/TNFR1 ELISA assay. Compounds were tested with respect to TNF using a battery of previously reported assays (Melagraki et al., 2017).

### Osteoclast Differentiation and TRAP Staining

Bone marrow (BM) cells were collected after flushing out of femurs and tibiae, subjected to gradient purification using Ficoll-Paque (GE Healthcare), plated in 96-well plates at a density of 6 × 10^4^ cells per well and cultured in AMEM medium (GIBCO) containing 10% fetal bovine serum supplemented with 40 ng/ml RANKL (Peprotech) and 25 ng/ml M-CSF (R&D Systems) for 5 days (Douni et al., 2012). Compounds A11 and A25 were pre-incubated with RANKL at various concentrations from 1 to 10 μM in AMEM medium for 1 h at room temperature and then added to cell cultures that were replenished with fresh medium every 2 days. Osteoclasts were stained for tartrate-resistant acid phosphatase (TRAP) activity using a leukocyte acid phosphatase (TRAP kit) (Sigma–Aldrich).

### TRAP Activity Assay

In the TRAP activity assay, BM cells were plated in 96-well plates at a density of 6 × 10^4^ cells per well and cultured in AMEM medium (GIBCO) containing 10% fetal bovine serum supplemented with 40 ng/ml RANKL (Peprotech) and 25 ng/ml M-CSF (R&D Systems) for 4 days. Then, cells were lysed in ice-cold phosphate buffer containing 0.1% Triton X-100. Lysates were added to 96-well plates containing phosphatase substrate (*p*-nitrophenol phosphate) and 40 mM tartrate acid buffer and incubated at 37°C for 30 min. The reaction was then stopped with the addition of 0.5 N NaOH. Absorbance was measured at 405 nm on a micro-plate reader (Optimax, Molecular Devices). TRAP activity was normalized to total protein which was determined using the Bradford assay (Bio-Rad).

### MTT Viability Assay

Cytotoxicity was evaluated for BM cells using the 3-(4,5-dimethylthiazol-2-yl)-2,5-diphenyltetrazolium bromide (MTT) assay, which measures the ability of viable cells to reduce a soluble tetrazolium salt to an insoluble purple formazan precipitate. BM cells used for MTT assay were seeded at a density of 10^5^ cells/well in 96-well plates and incubated with A11 and A25 compounds for 48 h in AMEM containing 10% fetal bovine serum supplemented with 25 ng/ml M-CSF (R&D Systems). After removal of the medium, each well was incubated with 0.5 mg/ml MTT (Sigma–Aldrich) in AMEM serum-free medium at 37°C for 2 h. At the end of the incubation period, the medium was removed and the intracellular formazan was solubilised with 200 μl DMSO and quantified by reading the absorbance at 550 nm on a micro-plate reader (Optimax, Molecular Devices). Percentage of cell viability was calculated based on the absorbance measured relative to the absorbance of the untreated control.

### Molecular Dynamics with EnalosMD

Molecular dynamics simulations were performed with our in-house developed EnalosMD suite of programs (EnalosMD, NovaMechanics Ltd., 2018). A fully automated pipeline included the following steps of systems’ preparation, MD runs, and analyses:

(a) Initial model structures were constructed with AmberTools16 (Case et al., 2016). Missing TNF and RANKL residues were added with Modeller 9.10 (Sali and Blundell, 1993; Fiser et al., 2000). The ff14SB force field (Maier et al., 2015) was used for the protein atoms and the general AMBER force field (GAFF) (Wang et al., 2004) represented compounds A11 and A25. Geometry optimization and AM1-BCC (Jakalian et al., 2002) charge derivation for A11 and A25 were obtained with ANTECHAMBER (Wang et al., 2006). The AM1-BCC approach is based on a fast and effective parameterization scheme that reliably reproduces the more rigorous RESP charges (Xu et al., 2013).
(b) AMBER-generated topology and coordinate files were subjected to four 1000 ns-long, all-atom, unrestrained MD simulations with the GPU version of OpenMM 7 (Eastman et al., 2017). Simulations were performed for (i) A11–TNF, (ii) A25–TNF, (iii) A11–RANKL, and (iv) A25–RANKL complexes in explicit solvent (TIP3P water model) (Jorgensen et al., 1983) and at 300 K with the GPU version of OpenMM. Periodic boundary conditions were used with a cutoff distance of 10 Å, and the Particle Mesh Ewald (PME) method (Darden et al., 1993) was employed for the treatment of long-range interactions. A Langevin thermostat with collision frequency set at 2.0 ps^-1^ regulated the temperature (Izaguirre et al., 2001).
(c) Analysis of the results (RMSD, atomic fluctuations, and hydrogen bond calculations) was performed with the cpptraj version of AmberTools.

## Results and Discussion

The formation of the biologically active TNF homotrimer is prevented by direct TNF inhibitors, such as SPD304, through disruption of the TNF dimer binding to the third subunit (He et al., 2005; Davis and Colangelo, 2012). TNF–inhibitor interactions are hydrophobic and shape-driven, as the inhibitor structure needs to be large enough to interact with both subunits and to prevent binding of the third subunit to the TNF dimer. We *in silico* explored 3,573 NPs contained in MEGxp database using a structure-based docking approach. The crystal structure of TNF dimer with SPD304 (PDB code: 2AZ5) was used as the molecular model for our investigation and the compounds were docked into the protein–protein interface. Computational molecular docking studies were performed using MVD (Thomsen and Christensen, 2006). Based on the docking score and following meticulous visual inspection of the conformations, we generated a shortlist of the top 15 commercially available NPs for *in vitro* validation.

Our *in vitro* screening strategy included one of the most commonly used assays of TNF activity. This assay exploits the ability of TNF to induce death in the murine fibrosarcoma cell line L929 following sensitization by the transcription inhibitor actinomycin D. Functional inhibition of TNF by small molecules would result in reduction of the TNF-induced cytotoxicity.

**FIGURE 1:**
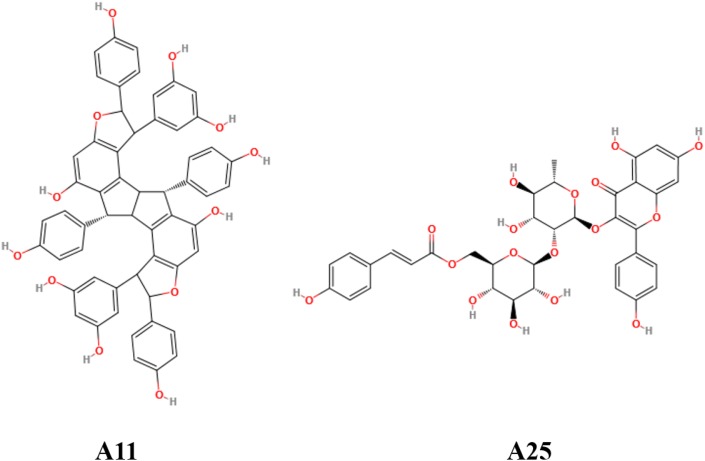
Chemical structures of the two most promising compounds (A11 and A25).

**FIGURE 2:**
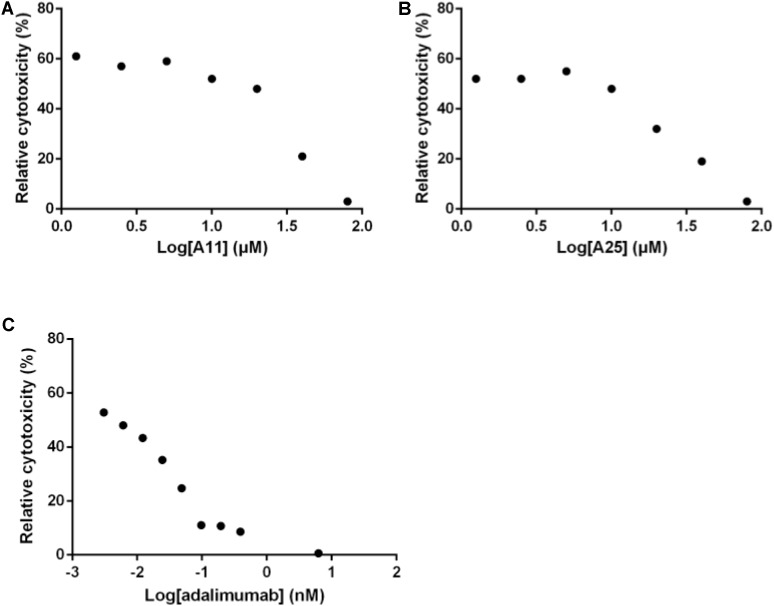
Inhibition of TNF-induced death in L929 cells. Increasing concentrations of A11 **(A)** or A25 **(B)** at 0.6–80 μM, and adalimumab **(C)** at 0.005–10 nM were used to pre-incubate recombinant human TNF (10 ng/ml) before addition to L929 cells for 18 h. Mean values (*n* = 3) relative to controls (TNF pre-incubated with DMSO or PBS in the adalimumab case) are shown. Data shown are representative of at least three experiments.

Out of the 15 prioritized NPs mentioned above, two emerged as the most promising ones based on *in vitro* testing. The action of these two NPs (designated A11 and A25; structures shown in **Figure 1**) was then further characterized. In dose–response experiments, the small molecules were shown to inhibit human TNF-driven death in L929 cells with an IC_50_ of 35 ± 3 μM (A11) and 33 ± 2 μM (A25). Both compounds were found to be minimally toxic in these cells (LC_50_ > 80 μM), in contrast to the published high toxicity of SPD304 (7.5 μM) (Melagraki et al., 2017). An already approved anti-TNF biologic, adalimumab (HUMIRA, Abbott Laboratories, IL, United States), was used as a positive control of the assay. Adalimumab is a human anti-TNF monoclonal antibody approved by the U.S. Food and Drug Administration (FDA, 2002) and by the European Medicines Agency (EMEA, 2003) for RA treatment. Adalimumab inhibits TNF-driven death in L929 cells with a low IC_50_ of 0.5 ± 0.1 nM, without showing any cytotoxicity (**Figure 2**).

**FIGURE 3:**
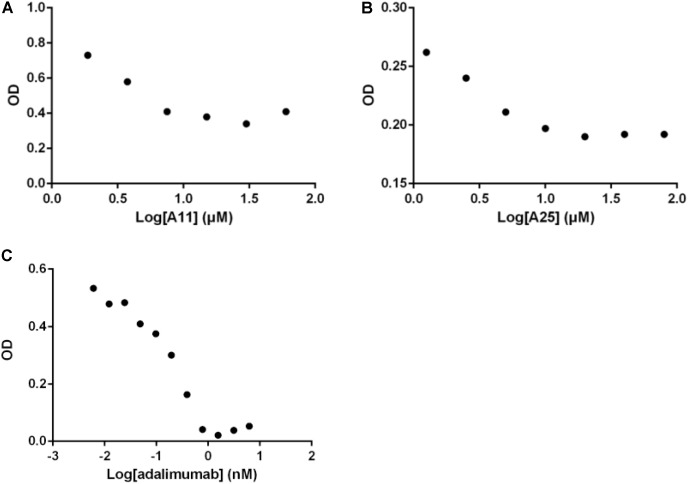
Disruption of the TNF/TNFR1 interaction. Increasing concentrations of A11 **(A)** or A25 **(B)** at 0.6–80 μM and adalimumab **(C)** at 0.005–10 nM were used to pre-incubate human TNF (10 ng/ml) before addition on a TNFR1 substrate. Binding was measured by ELISA. Mean values (*n* = 2) of one experiment, representative of at least three replicates are shown.

Having established that the selected products can obstruct the function of TNF, and given that TNF exerts its functions primarily through interacting with its receptor, TNFR1, an ELISA-based assay was used to quantify effects on this interaction. Both compounds significantly reduced binding of TNF to TNFR1, with an estimated IC_50_ of 3.3 ± 0.9 μM for A11 and 4.1 ± 1.7 μM for A25. Adalimumab was again used as a positive control eliminating the TNF-TNFR1 binding with a low IC_50_ of 0.2 nM (**Figure 3**).

The oligostilbenoid A11 (NP-003410, Ampelopsin H, (1R,2R,6R,6aR,7R,8R,12R,12aR)-1,7-Bis(3,5-dihydroxyphenyl)-2,6,8,12-tetrakis(4-hydroxyphenyl)-1,2,6,6a,7,8,12,12a-octahydrofuro[2″,3″:6′,7′]indeno[1′,2′:2,3]indeno [5,4-b]furan-5,11-diol) is an NP that has been isolated from *Parthenocissus tricuspidata* and the glycosyloxyflavone analog A25 (NP-008297, [(2R,3S,4S,5R,6S)-6-[(2S,3R,4R,5R,6S)-2-[5,7-dihydroxy-2-(4-hydroxyphenyl)-4-oxochromen-3-yl]oxy-4,5-dihydroxy-6-methyloxan-3-yl]oxy-3,4,5-trihydroxyoxan-2-yl]methyl(E)-3-(4-hydroxyphenyl)prop-2-enoate) is an NP that has been isolated from *Ginkgo biloba* (**Figure 1**). Except being isolated from natural sources, A11 can also be synthesized through a selective functionalization procedure as described by Rodrigues et al. (Rodrigues et al., 2016). Compounds A11 and A25 are promising PPI inhibitors as they both have large surface areas and are able to create many hydrophobic contacts at protein interfaces. Moreover, it has been observed that hydrophobic PPI hot-spot pockets tend to be excellent binders of small organic molecules, which combine a largely hydrophobic functionality with a secondary polar component (Guo et al., 2014). Indeed, the polar hydroxyl groups surrounding the hydrophobic core of A11 and A25 (**Figure 1**) constitute structures that are ideal binders to the concave hot-spot area of the protein (Mattos and Ringe, 1996; Shuker et al., 1996). It has been suggested that the ability of recognizing drug molecules (i.e., druggability) by a hot-spot pocket depends on the balance among total surface area, and polar/nonpolar contact areas (Hajduk et al., 2005; Cheng et al., 2007; Schmidtke and Barril, 2010).

In comparison to SPD304, NPs A11 and A25 are predicted by the molecular docking study to occupy a similar region in the binding pocket, and to be relatively hydrophobic and large enough to interact with residues from both subunits of the TNF dimer. Nonpolar residues are predominant in the binding site, which mainly includes glycine, leucine, and tyrosine. Only one HB interaction is observed between compound A25 and Tyr151. Both compounds appear to be situated more closely to subunit A than subunit B and are in close contact with the Leu120-Gly121-Gly122 β-strand of subunit A. The lack of salt bridges or extended HB interactions indicates the hydrophobic character of A11 and A25 binding as also observed with SPD304. The docked SPD304 conformation reproduced its crystal form, with an RMSD of 0.67 Å between the two structures. The docking score of SPD304 binding to TNF was calculated to be -171.08 (arbitrary units), and compounds A11 and A25 showed a binding score of -195.76 and -180.19, respectively, thus suggesting a strong interaction between the compounds and the TNF dimer. The high inhibitory potency of A11 and A25 against TNF was also indicated by our recently developed TNF model, released through the Enalos Cloud platform (Melagraki and Afantitis, 2014). After selecting the corresponding workflow within Enalos Cloud platform (Melagraki et al., 2017), both compounds were submitted and prediction results verified their activity. However, predictions fell out of the model’s domain of applicability as expected for these complex structures.

**FIGURE 4:**
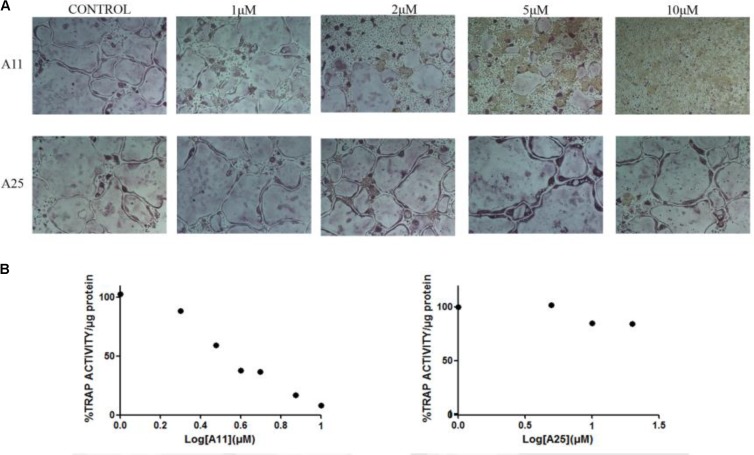
Effects of A11 and A25 on RANKL-induced osteoclastogenesis. **(A)** TRAP staining of osteoclastogenic cultures. BMMs were treated with A11 and A25 (1, 2, 5, and 10 μM) in the presence of RANKL (50 ng/ml) and M-CSF (25 ng/ml) for 5 days. **(B)** BMMs were treated with A11 (1, 2, 3, 4, 5, 7.5, and 10 μM) and A25 (1, 5, 10, and 20 μM) in the presence of RANKL (50 ng/ml) and M-CSF (25 ng/ml) for 4 days and cell lysates were measured for TRAP activity. % TRAP activity per microgram of total protein was expressed as a percentage of the untreated control. IC_50_ values are given as mean ± SEM from three independent experiments performed in duplicate.

**FIGURE 5:**
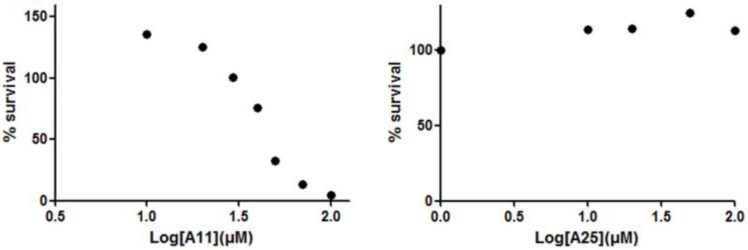
Effects of A11 and A25 on the viability of BMMs. BMMs were treated with 10–100 μM of compounds A11 and A25, respectively, in the presence of M-CSF (25 ng/ml) for 48 h. Cytotoxicity was assessed using a MTT colorimetric assay. Cell viability (%) was expressed as a percentage of the untreated control. LC_50_ values are given as mean ± SEM from three independent experiments performed in duplicate.

Receptor activator of nuclear factor kappa-B ligand, another TNF superfamily member, is the main regulator of osteoclast formation and bone resorption (Fuller et al., 1998). We evaluated the effect of various concentrations of A11 and A25 on RANKL-dependent osteoclast differentiation in a culture system of BM-derived monocyte/macrophages (BMMs) stimulated with RANKL (50 ng/ml) and M-CSF (25 ng/ml) for 5 days through evaluation of the TRAP activity, an osteoclast-specific enzyme. A11 fully suppressed RANKL-induced TRAP-positive osteoclast differentiation at 10 μM, whereas A25 was ineffective even at 20 μM (**Figure 4**). Moreover, using a quantitative assay that measures TRAP activity, A11 inhibited RANKL-induced osteoclastogenesis in a dose-dependent manner, displaying an IC_50_ of 3.42 ± 0.45 μM (**Figure 4B**). Furthermore, in order to exclude the possibility that inhibition of A11 on TRAP activity was due to cytotoxicity, the viability of BMMs was tested through the MTT assay. A11 displayed an LC_50_ of 44.76 ± 4.61 μM (**Figure 5**), suggesting that it affects osteoclastogenesis without interfering with cell viability. On the other hand, A25 had no effect either on osteoclastogenesis or BMM viability (LC_50_ > 100 μM) (**Figure 5**).

We subsequently investigated the binding of A11 to RANKL using the proposed molecular scaffolds in a structure-based approach. For this purpose, we employed the jFATCAT pairwise structure alignment algorithm (Ye and Godzik, 2003) to align the RANKL structure (PDB code: 1S55) to the crystal structure of TNF dimer with SPD304 (PDB code: 2AZ5). For our computational approach, we employed the murine RANKL model, which shares a 100% identity with human RANKL in the binding site, including residues Trp192, Tyr214, Asn275, Gly277, and Phe279. Also, RANKL shares a high degree of structural similarity with TNF as shown in Supplementary Figure S1. The binding conformations of both NPs and SPD304 are also depicted in the Supporting Information (Supplementary Figure S2). The docking methodology for RANKL systems was identical to the procedure followed for TNF complexes as described in the section “Materials and Methods.” The docking score of SPD304 binding to RANKL was calculated to be -159.712 and compounds A11 and A25 showed a binding score of -211.79 and -146.83, respectively. For A11, the computational analysis suggests a strong binding interaction with RANKL, which is in line with the experimental results.

**FIGURE 6:**
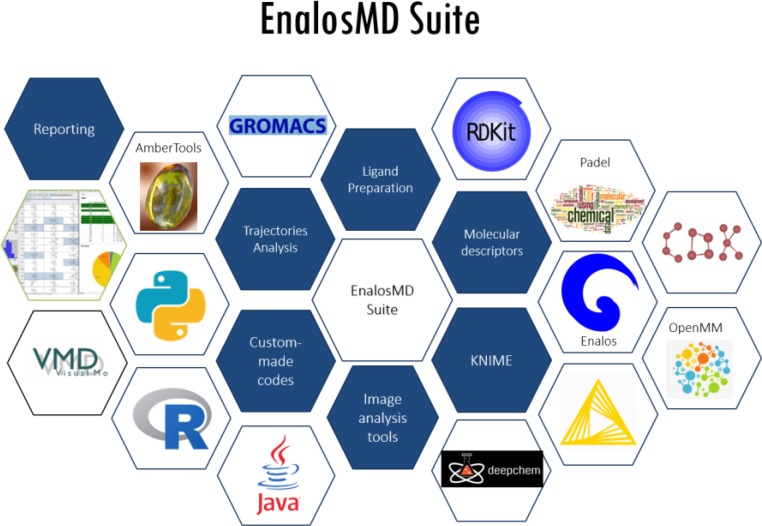
EnalosMD modules: High-throughput MD simulations are performed by optimally combining a variety of programs and functionalities.

Additionally, we employed our recently developed EnalosMD suite to perform extended MD simulations for A11 and A25 in complexes with TNF and RANKL. EnalosMD automates the preparation of any ligand-protein system and performs MD calculations in a way that minimal effort by the user is required. This application provides a powerful way to perform robust MD calculations with unprecedented speed and easiness regarding the construction of the initial model structure. Therefore, we carried out four 1000 ns-long MD runs to identify structural and energetic properties of the complexes that may further elucidate the mode of action of the two compounds. EnalosMD offers optimal performance by combining several computational programs and functionalities (**Figure 6**).

**FIGURE 7:**
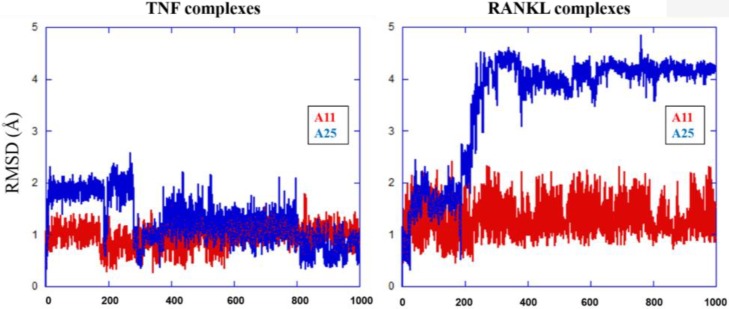
All-atom RMSD calculations for A11 and A25 in complexes with TNF and RANKL.

**FIGURE 8:**
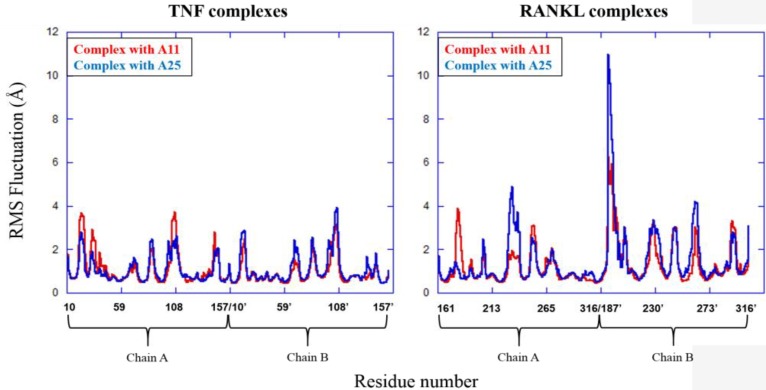
Atomic fluctuations for TNF and RANKL residues in complexes with A11 and A25.

**FIGURE 9:**
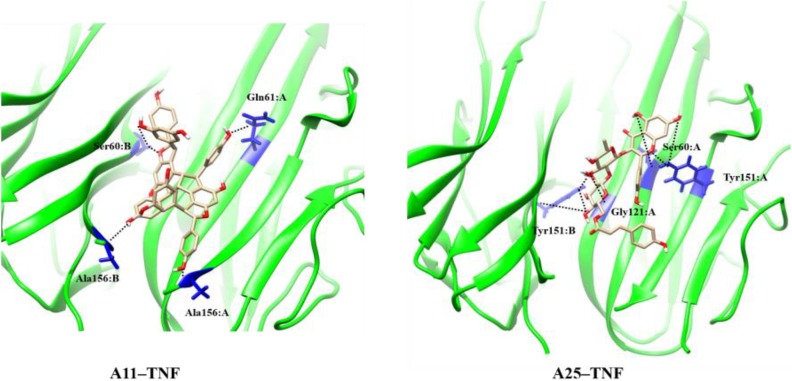
Main HB interactions between compounds and TNF.

**FIGURE 10:**
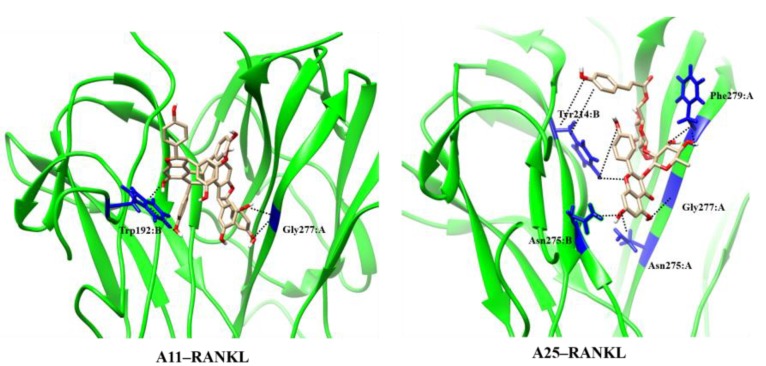
Main HB interactions between compounds and RANKL.

The MD results showed that protein structures early stabilized during the simulations in all complexes with RMSD values that do not exceed 3 and 4 Å in TNF and RANKL complexes, respectively (Supplementary Figure S3). A11 and A25 appear relatively stable into either protein’s cavity, with A25 showing only minor structural changes when bound to TNF after 200 ns (**Figure 7**). However, during the first 200–250 ns of A25–RANKL complex simulation, a noticeable conformational change of A25 stabilized the molecule in a new orientation with respect to the binding site of RANKL (**Figure 7**). This conformational change may have induced great flexibility to B chain terminal residues Tyr187–Asp189 as denoted by further fluctuation calculations (**Figure 8**). Therefore, the experimentally observed lower affinity of A25 against RANKL compared to A11 may be rationalized through the A25-induced destabilization of the terminal region of monomer B. Average conformations of A11 and A25 into their protein targets, along with protein residues that are involved in dominant HB interactions with the compounds are shown in **Figures 9**, **10**. The sole interaction between A25 and Tyr151, which was shown after docking calculations in TNF complex is also observed by the MD runs, however, it is complemented by three significant interactions from chain A (**Figure 9**).

## Conclusion

In summary, we have identified and validated experimentally the first plant-origin NPs that act as direct inhibitors of TNF by preventing the PPI between the dimer and the third subunit. Both NPs (A11 and A25) were shown to have IC_50_ values comparable to those of SPD304, but presented significantly reduced toxicity. Most importantly, A11 has been validated as the first NP dual inhibitor of TNF and RANKL. Both small molecules possess characteristics that are typical in potent PPI inhibitors, namely, large surface area and extended hydrophobic regions. Therefore, they can be explored as scaffolds representing NPs of plant origin in hit-to-lead optimization studies for the identification of direct TNF and/or RANKL inhibitors with improved pharmacological profiles and in the development of novel treatments for chronic inflammatory and autoimmune diseases.

## Ethics Statement

All animal procedures were approved and carried out in strict accordance with the guidelines of the Institutional Animal Care and Use Committee and the Region of Attica Veterinarian Office.

## Author Contributions

AA and GK conceptualization, funding acquisition, methodology, project administration, and supervision. GM, EN, VR, DP, GL, ED, AA, and GK data curation, formal analysis, investigation, resources, validation, visualization, writing – original draft, and writing – review and editing. GM, GL, and AA software.

## Conflict of Interest Statement

GL and AA are affiliated with NovaMechanics Ltd., a drug design company. The remaining authors declare that the research was conducted in the absence of any commercial or financial relationships that could be construed as a potential conflict of interest.

## References

[B1] Alzani R. Corti A. Grazioli L. Cozzi E. Ghezzi P. Marcucci F. (1993). Suramin induces deoligomerization of human tumor necrosis factor alpha. *J. Biol. Chem.* 268 12526–12529. 8509393

[B2] Apostolaki M. Armaka M. Victoratos P. Kollias G. (2010). Cellular mechanisms of TNF function in models of inflammation and autoimmunity. *Curr. Dir. Autoimmun.* 11 1–26. 10.1159/000289195 20173385

[B3] Arkin M. R. Tang Y. Wells J. A. (2014). Small-molecule inhibitors of protein-protein interactions: progressing toward the reality. *Chem. Biol.* 21 1102–1114. 10.1016/j.chembiol.2014.09.001 25237857 PMC4179228

[B4] Arkin M. R. Wells J. A. (2004). Small-molecule inhibitors of protein–protein interactions: progressing towards the dream. *Nat. Rev. Drug Discov.* 3 301–317. 10.1038/nrd1343 15060526

[B5] Baig M. H. Ahmad K. Roy S. Ashraf J. M. Adil M. Siddiqui M. H. (2016). Computer aided drug design: success and limitations. *Curr. Pharm. Des.* 22 572–581. 10.2174/1381612822666151125000550 26601966

[B6] Bakail M. Ochsenbein F. (2016). Targeting protein–protein interactions, a wide open field for drug design. *C. R. Chim.* 19 19–27. 10.1016/j.crci.2015.12.004

[B7] Basith S. Cui M. Macalino S. J. Y. Park J. Clavio N. A. B. Kang S. (2018). Exploring G protein-coupled receptors (GPCRs) ligand space via cheminformatics approaches: impact on rational drug design. *Front. Pharmacol.* 9:128. 10.3389/fphar.2018.00128 29593527 PMC5854945

[B8] Basse M. J. Betzi S. Bourgeas R. Bouzidi S. Chetrit B. Hamon V. (2013). 2P2Idb: a structural database dedicated to orthosteric modulation of protein–protein interactions. *Nucleic Acids Res.* 41 D824–D827. 10.1093/nar/gks1002 23203891 PMC3531195

[B9] Beutler B. Mahoney J. Le Trang N. Pekala P. Cerami A. (1985). Purification of cachectin, a lipoprotein lipase-suppressing hormone secreted by endotoxin-induced RAW 264.7 cells. *J. Exp. Med.* 161 984–995. 10.1084/jem.161.5.984 3872925 PMC2187615

[B10] Blevitt J. M. Hack M. D. Herman K. L. Jackson P. F. Krawczuk P. J. Lebsack A. D. (2017). Structural basis of small-molecule aggregate induced inhibition of a protein–protein interaction. *J. Med. Chem.* 60 3511–3517. 10.1021/acs.jmedchem.6b01836 28300404

[B11] Case D. A. Betz R. M. Cerutti D. S. Cheatham T. E. I Darden T. A. Duke R. E. (2016). *AMBER 2016.* San Francisco, CA: University of California.

[B12] Chan D. S.-H. Lee H.-M. Yang F. Che C.-M. Wong C. C. L. Abagyan R. (2010). Structure-based discovery of natural-product-like TNF-α inhibitors. *Angew. Chem. Int. Ed.* 49 2860–2864. 10.1002/anie.200907360 20235259 PMC4162403

[B13] Chaudhari K. Rizvi S. Syed B. A. (2016). Rheumatoid arthritis: current and future trends. *Nat. Rev. Drug Discov.* 15 305–306. 10.1038/nrd.2016.21 27080040

[B14] Cheng A. C. Coleman R. G. Smyth K. T. Cao Q. Soulard P. Caffrey D. R. (2007). Structure-based maximal affinity model predicts small-molecule druggability. *Nat. Biotechnol.* 25 71–75. 10.1038/nbt1273 17211405

[B15] Darden T. York D. Pedersen L. (1993). Particle mesh ewald: an N⋅log(N) method for ewald sums in large systems. *J. Chem. Phys.* 98 10089–10092. 10.1063/1.464397

[B16] David C. F. (2012). Small-molecule inhibitors of protein-protein interactions: how to mimic a protein partner. *Curr. Pharm. Des.* 18 4679–4684. 10.2174/138161212802651634 22650256

[B17] Davis J. M. Colangelo J. (2012). Small-molecule inhibitors of the interaction between TNF and TNFR. *Future Med. Chem.* 5 69–79. 10.4155/fmc.12.192 23256814

[B18] Douni E. Kollias G. (1998). A critical role of the p75 tumor necrosis factor receptor (p75TNF-R) in organ inflammation independent of TNF, lymphotoxin α, or the p55TNF-R. *J. Exp. Med.* 188 1343–1352. 10.1084/jem.188.7.1343 9763613 PMC2212501

[B19] Douni E. Rinotas V. Makrinou E. Zwerina J. Penninger J. M. Eliopoulos E. (2012). A RANKL G278R mutation causing osteopetrosis identifies a functional amino acid essential for trimer assembly in RANKL and TNF. *Hum. Mol. Genet.* 21 784–798. 10.1093/hmg/ddr510 22068587

[B20] Eastman P. Swails J. Chodera J. D. McGibbon R. T. Zhao Y. Beauchamp K. A. (2017). OpenMM 7: rapid development of high performance algorithms for molecular dynamics. *PLoS Comput. Biol.* 13:e1005659. 10.1371/journal.pcbi.1005659 28746339 PMC5549999

[B21] Elliott M. J. Maini R. N. Feldmann M. Long-Fox A. Charles P. Katsikis P. (1993). Treatment of rheumatoid arthritis with chimeric monoclonal antibodies to tumor necrosis factor alpha. *Arthritis Rheum.* 36 1681–1690. 10.1002/art.1780361206 8250987

[B22] EnalosMD NovaMechanics Ltd. (2018). Available at: http://enalosmd.novamechanics.com

[B23] Fiser A. Do R. K. Sali A. (2000). Modeling of loops in protein structures. *Protein Sci.* 9 1753–1773. 10.1110/ps.9.9.1753 11045621 PMC2144714

[B24] Fuller J. C. Burgoyne N. J. Jackson R. M. (2009). Predicting druggable binding sites at the protein–protein interface. *Drug Discov. Today* 14 155–161. 10.1016/j.drudis.2008.10.009 19041415

[B25] Fuller K. Wong B. Fox S. Choi Y. Chambers T. J. (1998). TRANCE is necessary and sufficient for osteoblast-mediated activation of bone resorption in osteoclasts. *J. Exp. Med.* 188 997–1001. 10.1084/jem.188.5.997 9730902 PMC2213394

[B26] Guerois R. Nielsen J. E. Serrano L. (2002). Predicting changes in the stability of proteins and protein complexes: a study of more than 1000 mutations. *J. Mol. Biol.* 320 369–387. 10.1016/S0022-2836(02)00442-4 12079393

[B27] Guharoy M. Pal A. Dasgupta M. Chakrabarti P. (2011). PRICE (PRotein interface conservation and energetics): a server for the analysis of protein–protein interfaces. *J. Struct. Funct. Genomics* 12 33–41. 10.1007/s10969-011-9108-0 21519818

[B28] Guo W. Wisniewski J. A. Ji H. (2014). Hot spot-based design of small-molecule inhibitors for protein–protein interactions. *Bioorg. Med. Chem. Lett.* 24 2546–2554. 10.1016/j.bmcl.2014.03.095 24751445

[B29] Hajduk P. J. Huth J. R. Fesik S. W. (2005). Druggability indices for protein targets derived from NMR-based screening data. *J. Med. Chem.* 48 2518–2525. 10.1021/jm049131r 15801841

[B30] He M. M. Smith A. S. Oslob J. D. Flanagan W. M. Braisted A. C. Whitty A. (2005). Small-molecule inhibition of TNF-α. *Science* 310 1022–1025. 10.1126/science.1116304 16284179

[B31] Hwang H. Vreven T. Janin J. Weng Z. (2010). Protein-protein docking benchmark version 4.0. *Proteins* 78 3111–3114. 10.1002/prot.22830 20806234 PMC2958056

[B32] Izaguirre J. A. Catarello D. P. Wozniak J. M. Skeel R. D. (2001). Langevin stabilization of molecular dynamics. *J. Chem. Phys.* 114 2090–2098. 10.1063/1.1332996

[B33] Jakalian A. Jack D. B. Bayly C. I. (2002). Fast, efficient generation of high-quality atomic charges. AM1-BCC model: II. Parameterization and validation. *J Comput. Chem.* 23 1623–1641. 10.1002/jcc.10128 12395429

[B34] Jones S. Thornton J. M. (1996). Principles of protein-protein interactions. *Proc. Natl. Acad. Sci. U.S.A.* 93 13–20. 10.1073/pnas.93.1.13 8552589 PMC40170

[B35] Jorgensen W. L. Chandrasekhar J. Madura J. D. Impey R. W. Klein M. L. (1983). Comparison of simple potential functions for simulating liquid water. *J. Chem. Phys.* 79 926–935. 10.1063/1.445869

[B36] Keffer J. Probert L. Cazlaris H. Georgopoulos S. Kaslaris E. Kioussis D. (1991). Transgenic mice expressing human tumour necrosis factor: a predictive genetic model of arthritis. *EMBO J.* 10 4025–4031. 1721867 10.1002/j.1460-2075.1991.tb04978.x PMC453150

[B37] Kollias G. Douni E. Kassiotis G. Kontoyiannis D. (1999). On the role of tumor necrosis factor and receptors in models of multiorgan failure, rheumatoid arthritis, multiple sclerosis and inflammatory bowel disease. *Immunol. Rev.* 169 175–194. 10.1111/j.1600-065X.1999.tb01315.x 10450517

[B38] Kollias G. Kontoyiannis D. (2002). Role of TNF/TNFR in autoimmunity: specific TNF receptor blockade may be advantageous to anti-TNF treatments. *Cytokine Growth Factor Rev.* 13 315–321. 10.1016/S1359-6101(02)00019-9 12220546

[B39] Kollman P. A. Massova I. Reyes C. Kuhn B. Huo S. Chong L. (2000). Calculating structures and free energies of complex molecules: combining molecular mechanics and continuum models. *Acc. Chem. Res.* 33 889–897. 10.1021/ar000033j 11123888

[B40] Kortemme T. Kim D. E. Baker D. (2004). Computational alanine scanning of protein-protein interfaces. *Sci. STKE* 2004:pl2. 10.1126/stke.2192004pl2 14872095

[B41] Kouadio J.-L. K. Horn J. R. Pal G. Kossiakoff A. A. (2005). Shotgun alanine scanning shows that growth hormone can bind productively to its receptor through a drastically minimized interface. *J. Biol. Chem.* 280 25524–25532. 10.1074/jbc.M502167200 15857837

[B42] Kozakov D. Grove L. E. Hall D. R. Bohnuud T. Mottarella S. E. Luo L. (2015a). The FTMap family of web servers for determining and characterizing ligand-binding hot spots of proteins. *Nat. Protoc.* 10 733–755. 10.1038/nprot.2015.043 25855957 PMC4762777

[B43] Kozakov D. Hall D. R. Jehle S. Luo L. Ochiana S. O. Jones E. V. (2015b). Ligand deconstruction: why some fragment binding positions are conserved and others are not. *Proc. Natl. Acad. Sci. U.S.A.* 112 E2585–E2594. 10.1073/pnas.1501567112 25918377 PMC4443342

[B44] Lesney M. S. (2004). Nature’s Pharmaceuticals. Natural products from plants remain at the core of modern medicinal chemistry. *Todays Chem. Work* 13 27–32.

[B45] Leung C.-H. Chan D. S.-H. Kwan M. H.-T. Cheng Z. Wong C.-Y. Zhu G.-Y. (2011). Structure-based repurposing of FDA-approved drugs as TNF-α inhibitors. *ChemMedChem* 6 765–768. 10.1002/cmdc.201100016 21365767

[B46] Leung C.-H. Zhong H.-J. Yang H. Cheng Z. Chan D. S.-H. Ma V. P.-Y. (2012). A metal-based inhibitor of tumor necrosis factor-α. *Angew. Chem. Int. Ed.* 51 9010–9014. 10.1002/anie.201202937 22807261

[B47] Lima M. N. N. Melo-Filho C. C. Cassiano G. C. Neves B. J. Alves V. M. Braga R. C. (2018). QSAR-driven design and discovery of novel compounds with antiplasmodial and transmission blocking activities. *Front. Pharmacol.* 9:146. 10.3389/fphar.2018.00146 29559909 PMC5845645

[B48] Lise S. Buchan D. Pontil M. Jones D. T. (2011). Predictions of hot spot residues at protein-protein interfaces using support vector machines. *PLoS One* 6:e16774. 10.1371/journal.pone.0016774 21386962 PMC3046169

[B49] Lo C. H. Vunnam N. Lewis A. Chiu T.-L. Brummel B. Schaaf T. (2017). Inhibition of tumor necrosis factor receptor 1 signaling by small molecules. *FASEB J.* 31 611–619. 10.1096/fasebj.31.1_supplement.609.11

[B50] London N. Raveh B. Schueler-Furman O. (2013). Druggable protein–protein interactions – from hot spots to hot segments. *Curr. Opin. Chem. Biol.* 17 952–959. 10.1016/j.cbpa.2013.10.011 24183815

[B51] Maier J. A. Martinez C. Kasavajhala K. Wickstrom L. Hauser K. E. Simmerling C. (2015). ff14SB: improving the accuracy of protein side chain and backbone parameters from ff99SB. *J. Chem. Theory Comput.* 11 3696–3713. 10.1021/acs.jctc.5b00255 26574453 PMC4821407

[B52] Mancini F. Toro C. M. Mabilia M. Giannangeli M. Pinza M. Milanese C. (1999). Inhibition of tumor necrosis factor-α (TNF-α)/ TNF-α receptor binding by structural analogues of suramin§. *Biochem. Pharmacol.* 58 851–859. 10.1016/S0006-2952(99)00150-1 10449196

[B53] Mattos C. Ringe D. (1996). Locating and characterizing binding sites on proteins. *Nat. Biotechnol.* 14 595–599. 10.1038/nbt0596-595 9630949

[B54] McGeary R. P. Bennett A. J. Tran Q. B. Cosgrove K. L. Ross B. P. (2008). Suramin: clinical uses and structure-activity relationships. *Mini Rev. Med. Chem.* 8 1384–1394. 10.2174/138955708786369573 18991754

[B55] Melagraki G. Leonis G. Ntougkos E. Rinotas V. Papaneophytou C. Mavromoustakos T. (2018). Current status and future prospects of small–molecule protein-protein interaction (PPI) inhibitors of tumor necrosis factor (TNF) and receptor activator of NF-κB ligand (RANKL). *Curr. Top. Med. Chem.* 18 1–13. 10.2174/1568026618666180607084430 29875003

[B56] Melagraki G. Ntougkos E. Rinotas V. Papaneophytou C. Leonis G. Mavromoustakos T. (2017). Cheminformatics-aided discovery of small-molecule Protein-Protein Interaction (PPI) dual inhibitors of Tumor Necrosis Factor (TNF) and Receptor Activator of NF-κB Ligand (RANKL). *PLoS Comput. Biol.* 13:e1005372. 10.1371/journal.pcbi.1005372 28426652 PMC5398486

[B57] Melagraki G. A. Afantitis A. (2014). Enalos InSilicoNano platform: an online decision support tool for the design and virtual screening of nanoparticles. *RSC Adv.* 4 50713–50725. 10.1039/C4RA07756C

[B58] Olsen N. J. Stein C. M. (2004). New drugs for rheumatoid arthritis. *N. Engl. J. Med.* 350 2167–2179. 10.1056/NEJMra032906 15152062

[B59] Perkins J. R. Diboun I. Dessailly B. H. Lees J. G. Orengo C. (2010). Transient protein-protein interactions: structural, functional, and network properties. *Structure* 18 1233–1243. 10.1016/j.str.2010.08.007 20947012

[B60] Rajamani D. Thiel S. Vajda S. Camacho C. J. (2004). Anchor residues in protein–protein interactions. *Proc. Natl. Acad. Sci. U.S.A.* 101 11287–11292. 10.1073/pnas.0401942101 15269345 PMC509196

[B61] Reutlinger M. Rodrigues T. Schneider P. Schneider G. (2014). Multi-objective molecular de novo design by adaptive fragment prioritization. *Angew. Chem. Int. Ed.* 53 4244–4248. 10.1002/anie.201310864 24623390

[B62] Rodrigues T. Reker D. Schneider P. Schneider G. (2016). Counting on natural products for drug design. *Nat. Chem.* 8 531–541. 10.1038/nchem.2479 27219696

[B63] Sackett D. L. Sept D. (2009). Protein-protein interactions: making drug design second nature. *Nat. Chem.* 1 596–597. 10.1038/nchem.427 21378947

[B64] Sali A. Blundell T. L. (1993). Comparative protein modelling by satisfaction of spatial restraints. *J. Mol. Biol.* 234 779–815. 10.1006/jmbi.1993.1626 8254673

[B65] Schmidtke P. Barril X. (2010). Understanding and predicting druggability. A high-throughput method for detection of drug binding sites. *J. Med. Chem.* 53 5858–5867. 10.1021/jm100574m 20684613

[B66] Sfikakis P. P. (2010). The first decade of biologic TNF antagonists in clinical practice: lessons learned, unresolved issues and future directions. *Curr. Dir. Autoimmun.* 11 180–210. 10.1159/000289205 20173395

[B67] Sheng C. Dong G. Miao Z. Zhang W. Wang W. (2015). State-of-the-art strategies for targeting protein-protein interactions by small-molecule inhibitors. *Chem. Soc. Rev.* 44 8238–8259. 10.1039/C5CS00252D 26248294

[B68] Shuker S. B. Hajduk P. J. Meadows R. P. Fesik S. W. (1996). Discovering high-affinity ligands for proteins: SAR by NMR. *Science* 274 1531–1534. 10.1126/science.274.5292.1531 8929414

[B69] Smith M. C. Gestwicki J. E. (2012). Features of protein-protein interactions that translate into potent inhibitors: topology, surface area and affinity. *Expert Rev. Mol. Med.* 14:e16. 10.1017/erm.2012.10 22831787 PMC3591511

[B70] Sun H. Yost G. S. (2008). Metabolic activation of a novel 3-substituted indole-containing TNF-α inhibitor: dehydrogenation and inactivation of CYP3A4. *Chem. Res. Toxicol.* 21 374–385. 10.1021/tx700294g 18095656

[B71] Thomsen R. Christensen M. H. (2006). MolDock: a new technique for high-accuracy molecular docking. *J. Med. Chem.* 49 3315–3321. 10.1021/jm051197e 16722650

[B72] Tuncbag N. Keskin O. Gursoy A. (2010). HotPoint: hot spot prediction server for protein interfaces. *Nucleic Acids Res.* 38(Suppl. 2), W402–W406. 10.1093/nar/gkq323 20444871 PMC2896123

[B73] Wang J. Wang W. Kollman P. Case D. (2006). Automatic atom type and bond type perception in molecular mechanical calculations. *J. Mol. Graph. Model.* 25 247–260. 10.1016/j.jmgm.2005.12.005 16458552

[B74] Wang J. Wolf R. Caldwell J. Kollman P. Case D. (2004). Development and testing of a general amber force field. *J. Comput. Chem.* 25 1157–1174. 10.1002/jcc.20035 15116359

[B75] Wilson A. J. (2009). Inhibition of protein-protein interactions using designed molecules. *Chem. Soc. Rev.* 38 3289–3300. 10.1039/b807197g 20449049

[B76] Xu L. Sun H. Li Y. Wang J. Hou T. (2013). Assessing the performance of MM/PBSA and MM/GBSA methods. 3. The impact of force fields and ligand charge models. *J. Phys. Chem. B* 117 8408–8421. 10.1021/jp404160y 23789789

[B77] Ye Y. Godzik A. (2003). Flexible structure alignment by chaining aligned fragment pairs allowing twists. *Bioinformatics* 19(Suppl. 2), ii246–ii255. 10.1093/bioinformatics/btg1086 14534198

[B78] Zheng S. Jiang M. Zhao C. Zhu R. Hu Z. Xu Y. (2018). e-Bitter: bitterant prediction by the consensus voting from the machine-learning methods. *Front. Chem.* 6:82. 10.3389/fchem.2018.00082 29651416 PMC5885771

