# Supplementary material for: In Silico Discovery of Plant-Origin Natural Product Inhibitors of Tumor Necrosis Factor (TNF) and Receptor Activator of NF-κB Ligand (RANKL)
Source: Front Pharmacol. 2018 Jul 25;9:800. doi: 10.3389/fphar.2018.00800 (PMC6068282; doi:10.3389/fphar.2018.00800)
Supplement: Supplementary file 1 [file Data_Sheet_1.docx]

**Supporting Information**

***In Silico* Discovery of Plant–Origin Natural Product Inhibitors of Tumor Necrosis Factor (TNF) and Receptor Activator of NF-κB Ligand (RANKL)**

Georgia Melagraki^1^, Evangelos Ntougkos^2^, Dimitra Papadopoulou^2^, Vagelis Rinotas^2,4^, Georgios Leonis^3^, Eleni Douni^2,4^, Antreas Afantitis^2,3*^, George Kollias^2,5*^

^1^Hellenic Army Academy, Vari, Greece

^2^Division of Immunology, Biomedical Sciences Research Center 'Alexander Fleming', Vari, Greece

^3^NovaMechanics Ltd, Nicosia, Cyprus

^4^Department of Biotechnology, Agricultural University of Athens, Athens, Greece

^5^Department of Experimental Physiology, Medical School, National and Kapodistrian University of Athens, Athens, Greece


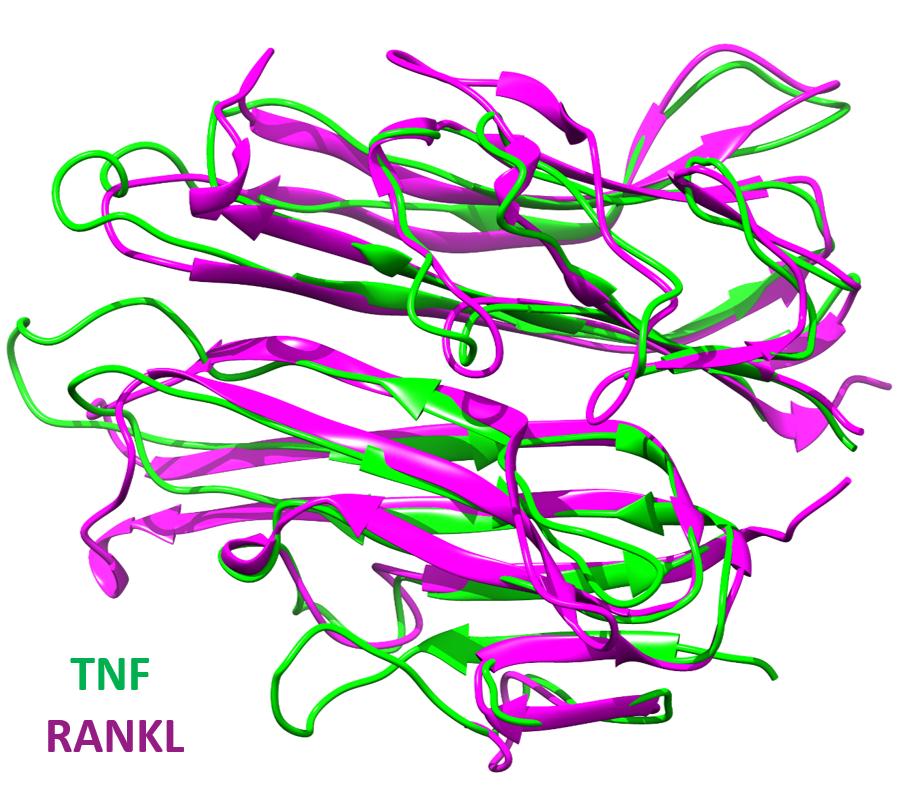


**Figure S1.** The superimposed dimeric structures of TNF and RANKL proteins.

**(a)**


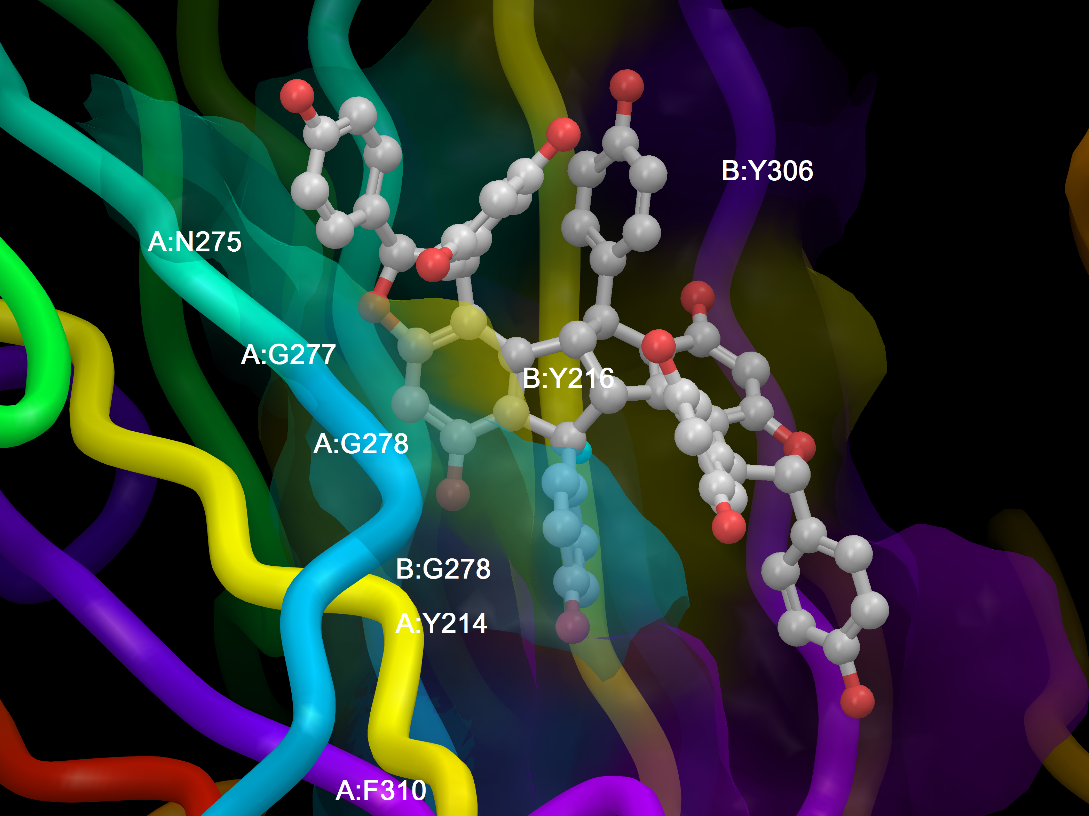


**(b)**


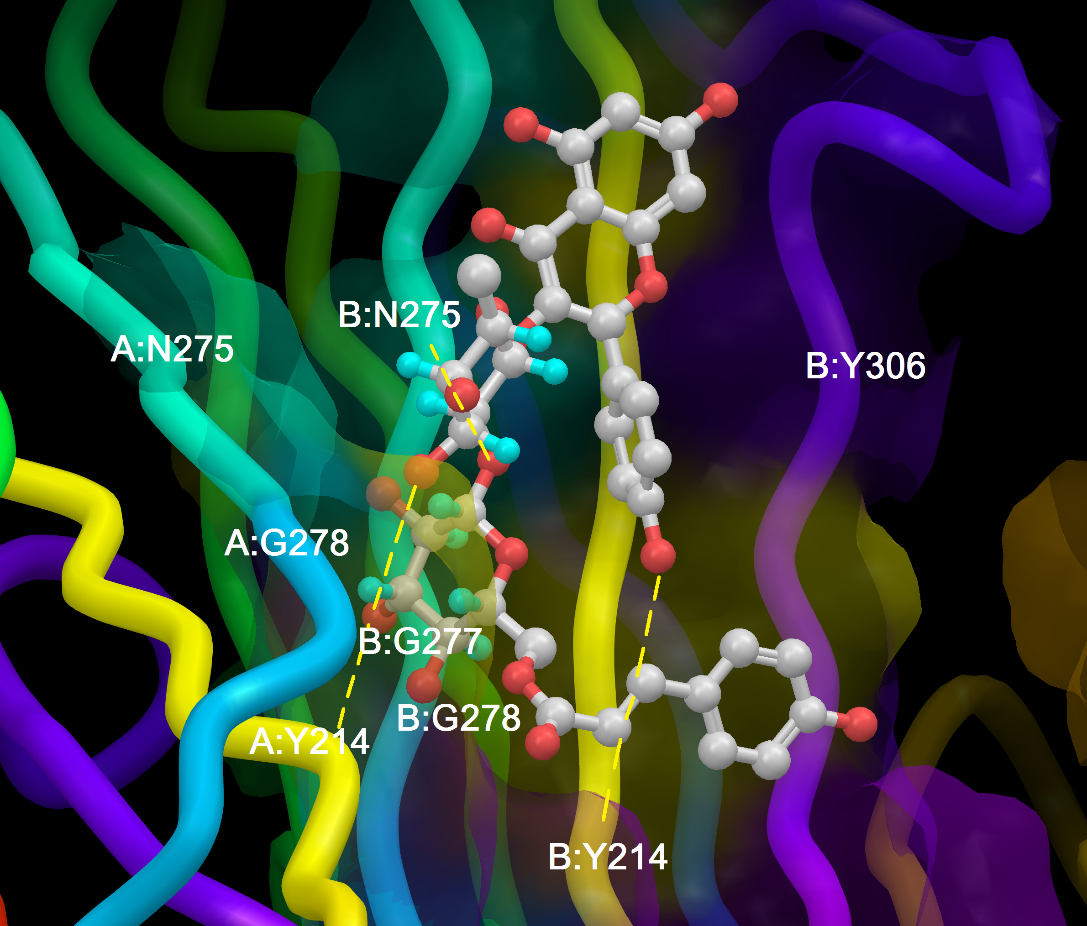


**(c)**


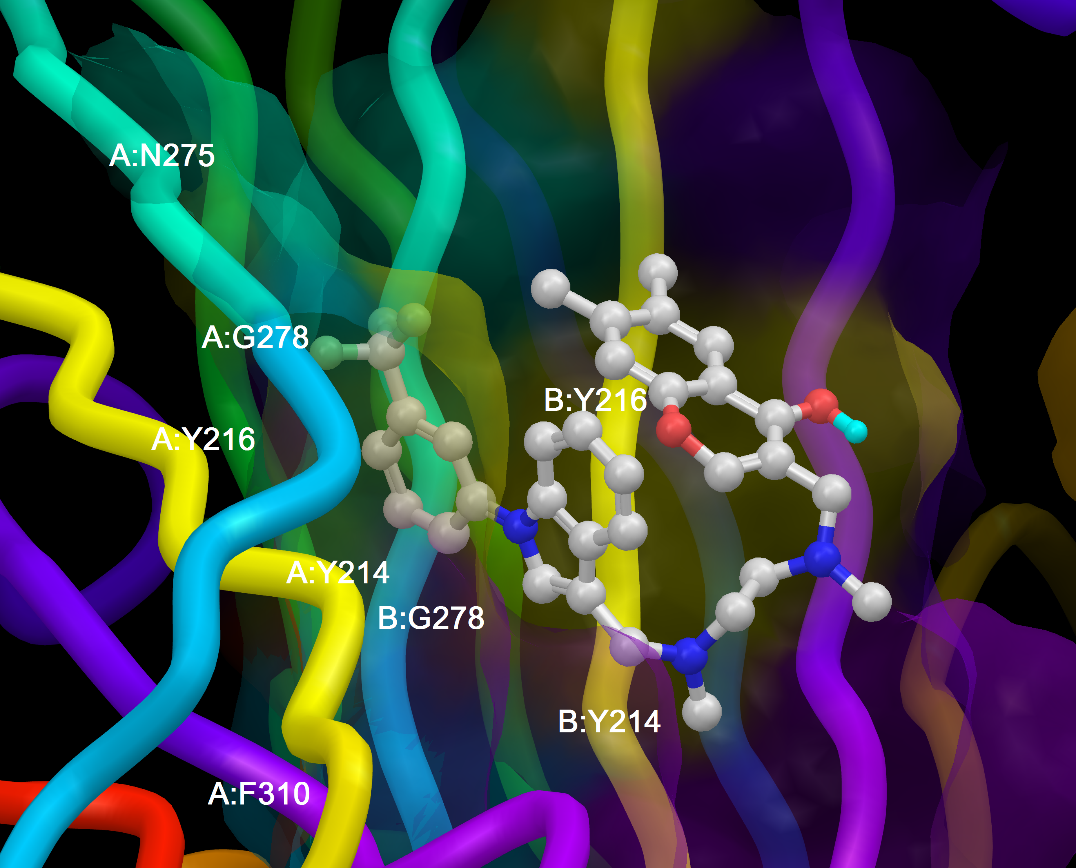


**Figure S2.** Binding conformations of: **(a)** A11, **(b)** A25 and **(c)** SPD304 to RANKL. Results obtained after molecular docking calculations.

**
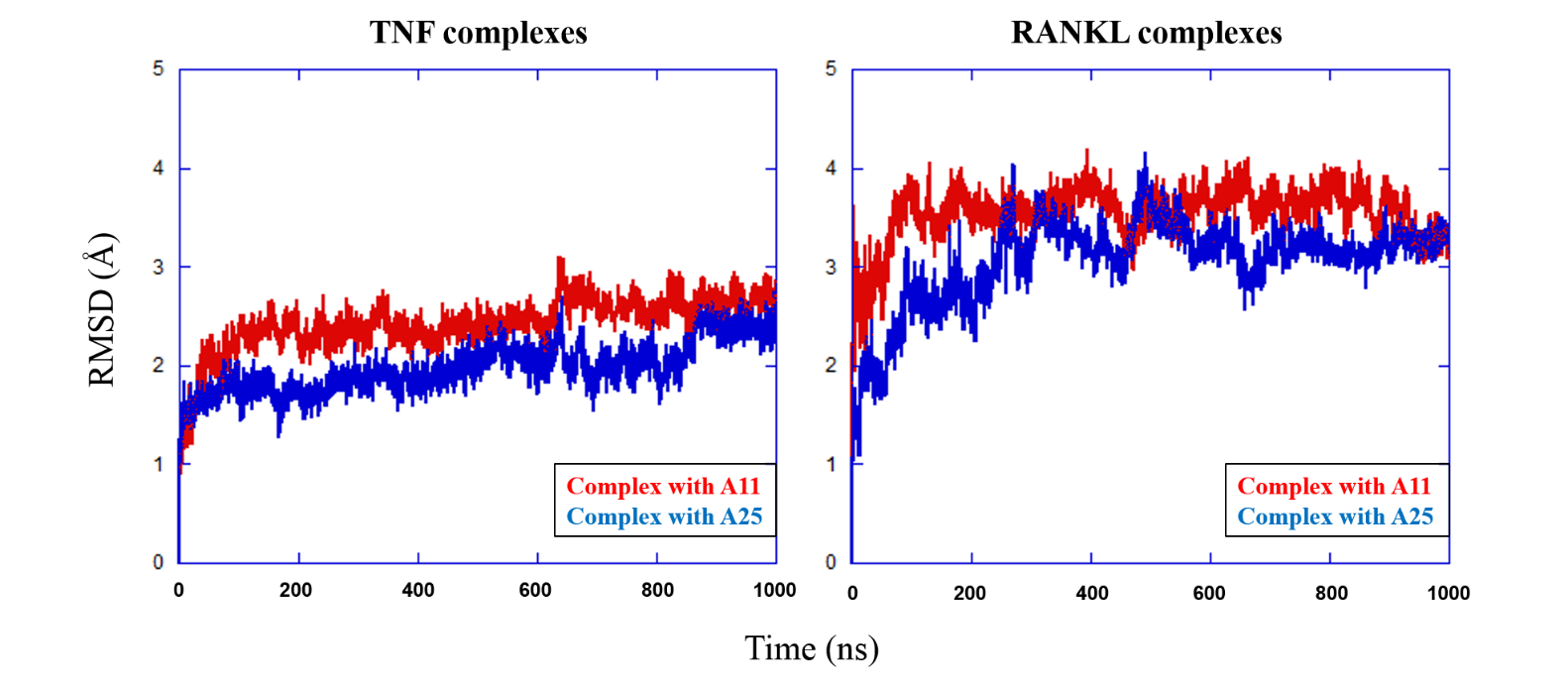
**

**Figure S3.** Cα RMSD calculations for TNF and RANKL in complexes with A11 and A25.
